# A new species of puddle frog from an unexplored mountain in southwestern Ethiopia (Anura, Phrynobatrachidae, *Phrynobatrachus*)

**DOI:** 10.3897/zookeys.824.31570

**Published:** 2019-02-12

**Authors:** Sandra Goutte, Jacobo Reyes-Velasco, Stephane Boissinot

**Affiliations:** 1 New York University Abu Dhabi, Saadiyat Island, Abu Dhabi, UAE New York University Abu Dhabi Abu Dhabi United Arab Emirates

**Keywords:** Bibita Mountain, Ethiopia, morphology, phylogenetic relationships, *Phrynobatrachusbibita* sp. n., taxonomy

## Abstract

A new species of *Phrynobatrachus* is described from the unexplored and isolated Bibita Mountain, southwestern Ethiopia, based on morphological characters and sequences of the mitochondrial rRNA16s. The new species can be distinguished from all its congeners by a small size (SVL = 16.8 ± 0.1 mm for males, 20.3 ± 0.9 mm for females), a slender body with long legs and elongated fingers and toes, a golden coloration, a completely hidden tympanum, and a marked canthus rostralis. The phylogenetic hypothesis based on 16s sequences places the new species as sister to the species group that includes *P.natalensis*, although it is morphologically more similar to other dwarf *Phrynobatrachus* species, such as the Ethiopian *P.minutus*.

## Introduction

**Figure 1.**
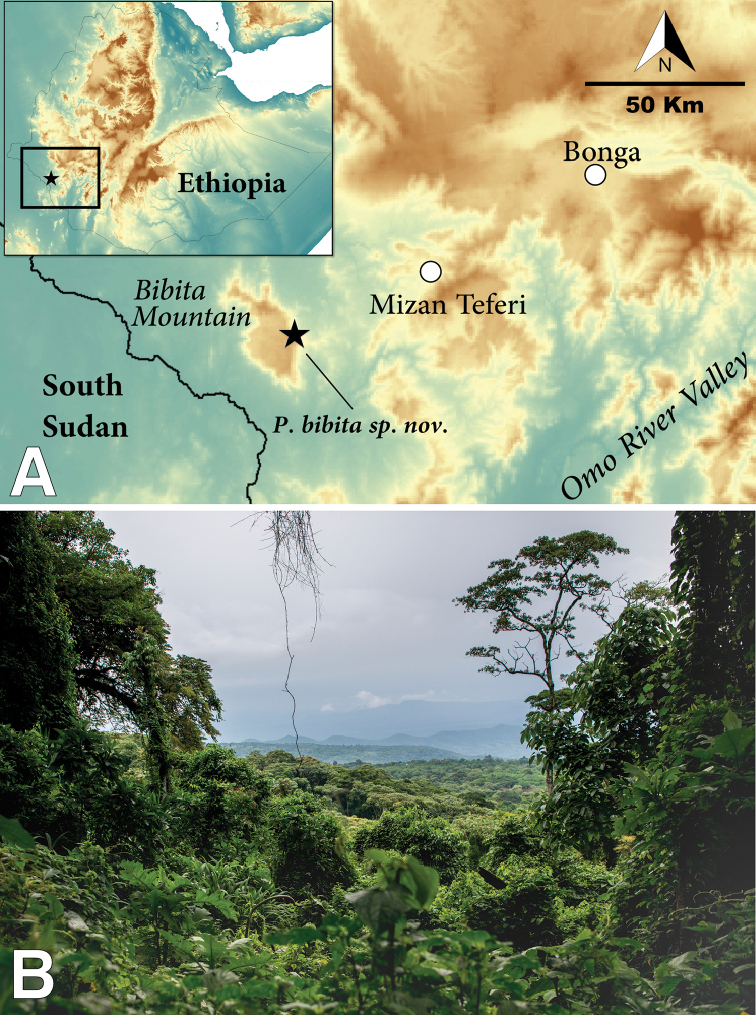
Type locality of *Phrynobatrachusbibita* sp. n. **A** Map of Ethiopia showing the location of Bibita Mountain **B** View from Bibita Mountain looking east, at an elevation of approximately 1900 m.

The highlands of Ethiopia are known for their high degree of diversity and endemism (Williams et al. 2004). Approximately half of all species of anurans (frogs and toads) of Ethiopia are endemic, including five endemic genera (Largen and Spawls 2010, Gower et al. 2013). Despite a recent increase in studies on the diversity of the Ethiopian fauna, the southwestern part of the country remains poorly studied, in part due to the difficulty in accessing the region. A small number of collections of amphibians have been obtained in this area, most of them from the vicinity of the towns of Bonga and Mizan Teferi (Figure 1A). Recent sampling campaigns conducted in the forests of the southwest revealed the existence of multiple undescribed taxa (Reyes-Velasco et al. 2018a, b). Additionally, the area harbors some of the last remaining forests in Ethiopia, giving it great potential for undiscovered diversity and making it of particular interest for taxonomists and conservationists.

While conducting fieldwork in the southwestern part of the country, we came across an undescribed species member of the genus *Phrynobatrachus*. This genus is one of the most species rich genera of African anurans, with 91 described and multiple undescribed taxa (Zimkus and Schick 2010, Frost 2018). This genus is widespread across sub-Saharan Africa. At least five species of *Phrynobatrachus* are found in Ethiopia: *P.bullans* Crutsinger, Pickersgill, Channing, and Moyer, 2004, *P.inexpectatus* Largen, 2001, *P.minutus* (Boulenger, 1895), *P.natalensis* (Smith, 1849), as well as *P.* sp. n. “Oromia” (Zimkus 2008, Largen and Spawls 2010, Zimkus et al. 2010, Frost 2018). The new species described here has multiple morphological characters that differentiate it from all other members of the genus, which is also supported by molecular evidence. Here, we describe the species as new to science.

## Materials and methods

### Taxonomic sampling

The mountain of Bibita in southwestern Ethiopia (6.8034N, 35.0602E), an isolated plateau located between the Gambela and the Southern Nations, Nationalities, and Peoples’ Regions, in the Bench Maji Zone, was explored (Figure 1). This mountain is approximately 50 km southwest of Mizan Teferi and ~18 km east from the border with South Sudan. Bibita Mountain is separated from the rest of the Ethiopian highlands by the lowlands formed by the tributaries of the Akobo River, including the Gilo River. This mountain is also sometimes referred to as Gurra Farda (Hundera and Deboch 2008), which translates to “donkey’s ears” in Oromo. On 16 and 17 June 2018, ten specimens of an undescribed species of *Phrynobatrachus* (two males, eight females) were collected, as well as multiple egg clutches from a pond in Bibita Mountain (6.82293N, 35.0938E; 1972 m a. s. l.). We photographed the specimens in situ, and took live photographs in captivity. We then euthanized them using a ventral application of 20% benzocaine gel (Chen and Comb 1999). After euthanasia, we took additional photographs of dorsal and ventral views of each specimen. We collected liver tissue samples from all specimens and egg clutches and stored them in RNAlater (ThermoFisher Scientific), and then fixed all specimens in 10% formalin for 48 hours and stored them in 70% Ethanol. All specimens are deposited at the Herpetology Collection of the Addis Ababa University, Addis Ababa, Ethiopia.

### Recordings of advertisement calls

A single calling male was recorded during the night of 17 June 2018 using a Sony Handycam camcorder. The soundtrack of the video was extracted using Adobe Premiere Pro and analyzed using Praat (Boersma and Weenink 2013). We analyzed two call bouts and fourteen notes produced by a single individual for dominant frequency, note duration, and interval inter-notes. We compared these calls to the advertisement calls of *Phrynobatrachusminutus* (recorded between Bonga and Jimma; 7.5350N, 36.5606E, 2216 m a. s. l.; SB233) and *P.natalensis* (east of Kibre Mengist; 5.8679N, 39.0490E, 1665 m a. s. l.; specimen not collected) which we recorded using a Marantz PMD661 MKII solid-state recorder and Sennheiser ME66 microphone. Unfortunately, calls for the other three *Phrynobatrachus* that occur in Ethiopia (*P.bullans*, *P.inexpectatus*, and *P.* sp. n. “Oromia” of Zimkus and Schick 2010) were not available for comparison.

### Morphological measurements

For each specimen of the type series, we used an SPI dial caliper (model #31-415-3, precision: 0.1 mm) to take the following measurements as defined in Watters et al. (2016):

**SVL** snout vent length,

**HL** head length,

**HW** head width,

**SL** snout length,

**NS** nostril-snout distance,

**ED** eye diameter,

**EN** eye-nostril distance,

**IOD** interorbital distance,

**IND** inter-narinal distance,

**ED** eye diameter,

**UEW** pper eyelid width,

**FLL** forearm length,

**HAL** hand length,

**FinDW** third finger disk width,

**THL** thigh length,

**TL** tibiofibula length,

**FL** foot length,

**Toe4DW** toe IV disk width,

**MTL** metatarsal tubercle length.

### DNA extraction, PCR amplification, and phylogenetic analyses

We extracted DNA from the liver tissue samples stored in RNAlater with the use of standard potassium acetate protocol, or with the use of a DNeasy Blood & Tissue Kit (Qiagen). We also extracted DNA from eggs collected on leaves where females and the amplected pair of the new species were found, to ensure that these belonged to the new species. We then measured DNA concentration for each one of the samples using a broad range kit in a Qubit fluorometer (Life Technologies). We amplified a fraction of the 16s rRNA mitochondrial gene with the use of the primers LX12SN1a (forward) and LX16S1Ra (reverse) of Zhang et al. (2013). We performed Polymerase Chain Reaction (PCR) in total volumes of 48 μl with the use of regular Taq (Invitrogen), with the following conditions: initial denaturation at 94 °C (two minutes), followed by 35 cycles consisting of a denaturation step at 94 °C (30 seconds), annealing step at 48 °C (30 seconds), and extension step at 72 °C (one minute). The final extension step consisted of one minute at 72 °C. We shipped the unpurified PCR products for sequencing at BGI Tech Solutions (Hong Kong).

We used the program Geneious v 9.1.6 (Biomatters Ltd., Auckland, NZ) to manually trim and edit the raw chromatograms. We included additional sequences of *Phrynobatrachus* from GenBank in order to infer the phylogenetic positions of the new material. We deposited all new sequences in GenBank (Suppl. material 1: Table S1). We aligned all sequences in MAFFT (Katoh et al. 2017) version 7, with the Q-INS-I option, which resulted in a final alignment of 589 bp. We then selected the best-fit model of nucleotide substitution in PartitionFinder v.1.1.1 (Lanfear et al. 2012). The model selected was the K2P + I + gamma. We performed Bayesian inference of phylogeny (BI) in MrBayes v 3.2.2 (Ronquist et al. 2012) in the CIPRES science gateway server (Miller et al. 2010). The Bayesian analysis consisted of four runs of 10^7^ generations each, with four chains (one cold and three heated), sampling every 1,000 generations. We used Tracer v1.6 (Drummond and Rambaut 2007) to confirm that independent runs had converged, based on the overlap in likelihood and parameter estimates among runs, as well as effective sample size (ESS) and Potential Scale Reduction Factor value estimates (PSRF). PSRF indicated that individual runs had converged by 10^5^ generations, so we discarded the first 25% of the runs as burn-in. We then annotated posterior probability values on the resulting topology in the program TreeAnnotator v 1.8.3 (Rambaut 2014).

## Systematic account

### Phrynobatrachus bibita

Taxon classificationAnimaliaAnuraPhrynobatrachidae

Goutte, Reyes-Velasco & Boissinot sp. n.

http://zoobank.org/3B046650-2B48-4740-9ED9-A29A4EC10A72

#### Material.

**Figure 2.**
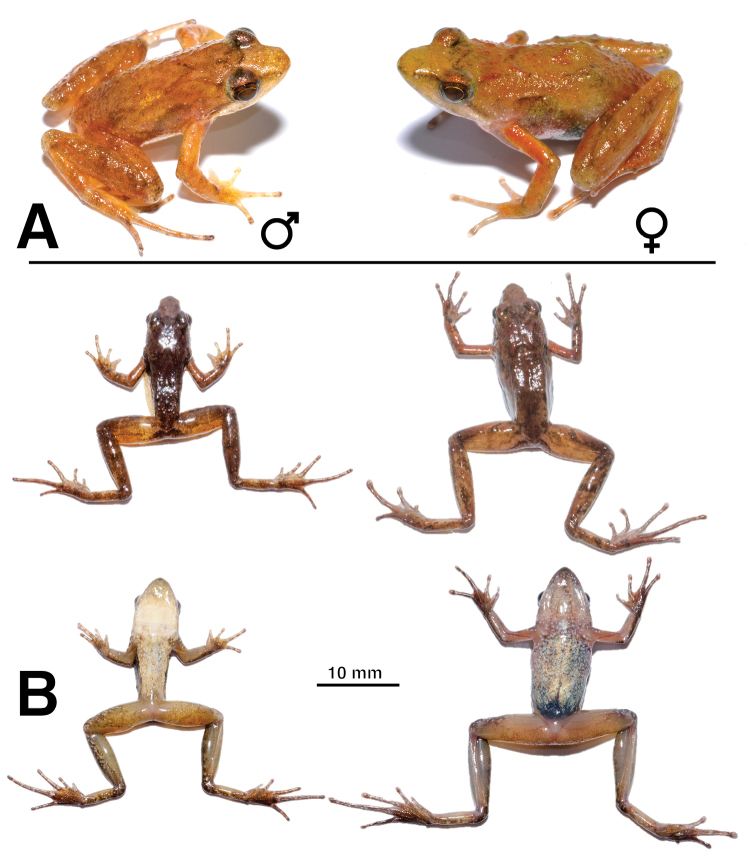
*Phrynobatrachusbibita* sp. n. **A** Live pictures of *P.bibita* sp. n. Male holotype (left; SB440) and female paratopotype (right; SB424) **B** Ventral and dorsal views of the same individuals, with male on the left and female on the right. Scale bar: 10 mm.

***Holotype.*** A male (SB440; Figure 2), collected on 17 June 2018, at night, in Bibita Mountain, southwestern Ethiopia (6.8229N, 35.0938E, datum = WGS84; 1972 m a.s.l.). The specimen was collected while in amplexus (female paratopotype SB439), on vegetation, in overgrown pond in primary forest. ***Paratopotypes.*** Nine specimens: eight females collected in the same pond as the holotype on 16 June 2018 (SB418-SB420, SB424-SB427) and on 17 June 2018 (SB439); another male (SB421) collected at night in the water (probably calling), at same locality as the holotype on 16 June 2018.

#### Diagnosis.

Small species (SVL = 16.8 ± 0.1 mm for males, 20.3 ± 0.9 mm for females) attributed to the genus *Phrynobatrachus* by the presence of tarsal and outer metatarsal tubercles (Suppl. material 2: Figure S1A). Body slender, with long legs (tibia length/SVL = 0.6 in both sexes) rather long snout for the genus and very elongated fingers (hand length/SVL = 0.3 in both sexes) and toes (foot length/SVL = 0.6 in both sexes) in comparison to its congeners. Webbing absent between fingers and minimal between toes. Tympanum not visible. Canthus rostralis marked and concave from nostril to eye. Snout pointed. Nostrils not visible from above. Eyelid spine absent. Throat of adult males white with light grey freckles on the anterior third, without any spinulae. Femoral glands hardly distinguishable but present in adult males. Two ridges in the scapular region and two short, oblique ridges behind the eyes. These four ridges may be all disjointed, the two scapular ridges may be jointed to form a chevron shape, or the ridges may be jointed laterally in an hourglass shape.

#### Comparisons.

This species can easily be distinguished from other Ethiopian *Phrynobatrachus*: the body is slenderer, the hind limbs, fingers and toes are longer than all four other described Ethiopian *Phrynobatrachus*, *P.bullans*, *P.natalensis*, *P.minutus*, and *P.inexpectatus*. Additionally, the tips of fingers and toes are more enlarged than in any of these species, particularly in females. It is similar in size as *P.minutus*, slightly larger than *P.inexpectatus* and much smaller than *P.bullans* and *P.natalensis*. It can be further distinguished from *P.natalensis* by a more marked canthus rostralis and a completely hidden tympanum. Adult males of *P.bibita* sp. n. can be distinguished from *P.bullans*, *P.natalensis*, *P.minutus*, and *P.inexpectatus* by the white coloration of their throat (Largen 2001, Crutsinger et al. 2004). The new species is also distinct from the three junior synonyms of *P.natalensis* described from Ethiopia: *Arthroleptisbottegi* Boulenger, 1895, described from Auta, Bale province, possesses a distinct tympanum, a rounded snout, and is much larger than *P.bibita* (SVL of female holotype = 31 mm vs. 20.3 ± 0.9 mm in female *P.bibita*). *Phrynobatrachussciangallarum* Scortecci, 1943, described from a presumably adult male from Murle, Gemu-Gofa province, differs from *P.bibita* by the presence of a considerably darkened throat and the lack of femoral glands. Additionally, it has more extensive foot webbing than *P.bibita* (Largen 2001). *Arthroleptis-Phrynobatrachuszavattarii* Scortecci, 1943, described from Caschei, Gemu-Gofa province, only differs from *P.sciangallarum* by the extent of the foot webbing, and all measurements of the type specimens fall within the range of *P.natalensis* (Largen 2001). Blackburn (2014) provided color photographs of the holotypes of these last two junior synonyms of *P.natalensis*. Based on those photographs, it appears that *P.bibita* has a slenderer head and more elongated fingers than these individuals. Finally, the type localities for these three junior synonyms of *P.natalensis* are in the southern part of the country, an area that is dry and at low elevation, in contrast with the habitat occupied by *P.bibita* sp. n.

Among Eastern African *Phrynobatrachus*, *P.bibita* sp. n. can be distinguished from *P.acridoides* (Cope, 1867), *P.auritus* Boulenger, 1900, *P.dendrobates* (Boulenger, 1919), *P.graueri* (Nieden, 1911), *P.keniensis* Barbour & Loveridge, 1928, *P.krefftii* Boulenger, 1909, *P.perpalmatus* Boulenger, 1898, *P.petropedetoides* Ahl, 1924, and *P.versicolor* Ahl, 1924, by a completely hidden tympanum (Cope 1867, Boulenger 1882, 1909, Razzetti and Msuya 2002, Pickersgill 2007). This species can further be distinguished from *P.auritus* Boulenger, 1900, *P.dendrobates*, *P.graueri*, *P.kinagopensis* Angel, 1924, *P.krefftii*, *P.mababiensis* FritzSimons, 1932, *P.parvulus* (Boulenger, 1905), *P.rouxi* (Nieden,1912), *P.scheffleri* (Nieden, 1911), and *P.ukigensis* Grandison & Howell, 1983, by the white and light grey coloration of adult males’ throat (Boulenger 1909, Angel 1924, FritzSimons 1932). The new species differs from *P.bullans*, *P.kakamikro* Schick, Zimkus, Channing, Köhler, & Lötters, 2010, and *P.perpalmatus* by the presence of femoral glands in adult males (Crutsinger et al. 2004, Pickersgill 2007, Schick et al. 2010). The new species is also larger than *P.pallidus*, *P.parvulus*, *P.scheffleri*, and *P.ungujae* Pickersgill, 2007 (Boulenger 1905, Pickersgill 2007, Schick et al. 2010), and possesses longer legs than *P.kakamikro* and *P.pallidus* Pickersgill, 2007, as well as longer feet than *P.pallidus* and *P.ungujae* (Pickersgill 2007, Schick et al. 2010). Finally, *P.bibita* sp. n. can be distinguished from *P.acridoides*, *P.bullans*, *P.mababiensis*, *P.natalensis*, *P.pallidus*, *P.ungujae*, and *P.versicolor* Ahl, 1924, by the absence of dark bars on the lower jaw (Cope 1867, Boulenger 1882, Fritzsimons 1932, Crutsinger et al. 2004, Pickersgill 2007).

#### Description of the holotype.

Adult male (Figure 2; Table 1), body slender, head longer than wide (HW/HL = 0.96), the snout is pointed and the canthus rostralis is marked and concave from nostril to eye. Tympanum not visible. Pupil horizontal. Eyelid spine absent. Maxillary teeth present, vomerine teeth absent. Tongue elongate, free for two thirds of its length, tip divided in two short lobes. Legs and feet long for the genus (TL/SVL = 0.6; FL/SVL = 0.6). Tarsal tubercle and outer metatarsal tubercle present, but inner metatarsal tubercle not distinguishable. Subarticular tubercles absent on the hands and small and barely distinguishable on the feet. Palmar tubercle absent. Fingers and toes elongated and their tips slightly swollen but not forming discs. First fingers shorter and swollen but nuptial pads absent. Fingers free of webbing. No webbing between toe I and II, and minimal webbing between the other toes. Finger formula: I<II<IV<III. Toe formula: I<II<V<III<IV. Skin of dorsum and dorsal side of limbs covered by small pointy asperities. Femoral glands hardly visible but present (Suppl. material 2: Figure S1B). Chevron-shaped gland in scapular region extending from above the shoulder girdle to approx. mid-body. Two rows of small warts form faint ridges between the eye and the scapular region but do not reach the chevron-shaped gland. The skin of the throat is not thin and loose as in many other *Phrynobatrachus* species and gular folds and spinulae are absent.

**Table 1.** Morphometric measurements of *Phrynobatrachusbibita* sp. n.

Specimen	SB 440	SB 421	SB 418	SB 419	SB 420	SB 424	SB 425	SB 426	SB 427	SB 439	Males (n = 2)	Females (n = 8)
Sex	♂	♂	♀	♀	♀	♀	♀	♀	♀	♀		
SVL	16.8	16.7	19.3	19.2	21.5	20.6	20.9	20.3	21.0	19.4	16.6 ± 0.1	20.3 ± 0.9
HW	5.5	5.7	5.9	6.2	6.8	6.2	6.5	6.5	7.3	5.9	5.6 ± 0.1	6.4 ± 0.5
HL	5.7	5.8	6.6	6.7	7.1	7.4	6.9	6.6	6.8	6.7	5.6 ± 0.1	6.9 ± 0.3
SL	2.4	2.7	2.8	3.0	3.3	3.3	3.2	3.1	3.0	2.8	2.6 ± 0.2	3.1 ± 0.2
NS	1.4	1.1	1.5	1.6	1.6	1.8	1.6	1.7	1.7	1.7	1.3 ± 0.2	1.7 ± 0.1
IND	2.0	1.8	2.1	2.2	2.4	2.3	2.3	2.4	2.1	2.2	1.9 ± 0.1	2.3 ± 0.1
EN	1.2	1.6	1.5	1.5	1.6	1.4	1.5	1.2	1.3	1.2	1.4 ± 0.3	1.4 ± 0.2
IOD	2.0	2.0	1.8	2.2	2.2	2.5	2.0	2.4	1.9	2.1	2.0 ± 0.0	2.1 ± 0.2
ED	2.5	1.8	2.0	2.3	2.0	2.3	2.3	2.1	2.1	2.2	2.2± 0.5	2.2 ± 0.1
UEW	1.8	1.3	1.4	1.8	1.5	1.9	1.6	1.5	1.5	1.8	1.6 ± 0.4	1.6 ± 0.2
FLL	3.2	3.7	3.5	4.1	3.8	4.6	4.0	3.9	4.0	3.6	3.5 ± 0.4	3.9 ± 0.3
HAL	5.1	5.0	5.4	5.5	6.0	7.5	6.5	5.8	6.5	6.1	5.1 ± 0.1	6.2 ± 0.7
FinDW	0.6	0.3	0.6	0.7	0.7	0.8	0.7	0.7	0.6	0.8	0.5 ± 0.2	0.7 ± 0.1
THL	8.0	8.0	8.9	9.1	9.6	9.7	9.6	9.6	9.1	9.4	8.0 ± 0.0	9.4 ± 0.3
TL	9.5	9.6	10.6	10.8	11.5	11.6	11.2	11.4	11.2	10.7	9.6 ± 0.1	11.1 ± 0.4
FL	10.5	9.5	12.7	11.7	11.3	12.2	11.5	11.5	11.0	11.4	10 ± 0.7	11.7 ± 0.5
Toe4DW	0.3	0.4	1.0	0.6	0.7	0.6	0.6	0.9	0.6	0.6	0.4 ± 0.1	0.7 ± 0.2
MTL	NA	0.6	NA	NA	0.7	0.4	1.1	NA	NA	0.7	–	–
TL/SVL	0.57	0.57	0.55	0.56	0.53	0.56	0.54	0.56	0.53	0.55	0.6 ± 0.0	0.6 ± 0.0
HW/HL	0.96	0.98	0.89	0.93	0.96	0.84	0.94	0.98	1.07	0.88	1.0 ± 0.0	0.9 ± 0.1
EN/IND	0.60	0.89	0.71	0.68	0.67	0.61	0.65	0.50	0.62	0.55	0.7 ± 0.1	0.6 ± 0.1
HW/SVL	0.33	0.34	0.31	0.32	0.32	0.30	0.31	0.32	0.35	0.30	0.3 ± 0.0	0.3 ± 0.0
FL/TL	1.11	0.99	1.20	1.08	0.98	1.05	1.03	1.01	0.98	1.07	1.1 ± 0.1	1.1 ± 0.1
FL/SVL	0.63	0.57	0.66	0.61	0.53	0.59	0.55	0.57	0.52	0.59	0.6 ± 0.0	0.6 ± 0.0
HL/SVL	0.34	0.35	0.34	0.35	0.33	0.36	0.33	0.33	0.32	0.35	0.3 ± 0.0	0.3 ± 0.0

Measurements for the male holotype and the paratopotypes, and average values ± standard deviations for males and females are given in millimeters. Abbreviations: SVL = snout vent length, HL = head length, HW = head width, SL = snout length, NS = nostril-snout distance, ED = eye diameter, EN = eye-nostril distance, IOD = interorbital distance, IND = inter-narinal distance, ED = eye diameter, UEW = upper eyelid width, FLL = forearm length, HAL = hand length, FinDW = third finger disk width, THL = thigh length, TL = tibiofibula length, FL = foot length, Toe4DW = Toe IV disk width, MTL = metatarsal tubercle length.

#### Coloration of holotype in life.

The body is golden (Figure 2A); a dark chevron is present in the scapular region underlining two short back ridges. A faint dark triangle is present between the eyes and a faint dark bar is present between the eye and the shoulder. The upper eyelids are darker than the rest of the head. Loreal region faintly darker than the rest of the head. Upper and lower jaws lack any blotching. The flanks and the sides of the head present numerous small white spots, extending to the upper lip below the eye. Iris dark gold with a golden ring around the iris interrupted by a black dot at the bottom of the iris. The thighs and legs are faintly and irregularly banded. The back of the thighs is yellowish with irregular coverage of melanophores. A dark triangle is present in the vent region. Ventral region is white molted with light grey. The throat is white, with the anterior third light grey with white freckles. Ventral side of the thighs and legs shows irregular melanophores and white spots.

#### Coloration of holotype in preservative.

After euthanasia, the individual’s coloration darkened notably (Figure 2B). In preservative, the dorsum presents a grey coloration.

#### Variation.

Morphometric variations of the type series are summarized in Table 1. Inner metatarsal tubercles are not visible in all individuals. Subarticular tubercles are more pronounced in some individuals. Webbing of the feet presents small variation, ranging from minimal to no webbing for toes I and II, and webbed to the first phalange for toes III to V. The dorsal and leg skin is mostly smooth with small warts more pronounced in some individuals. The two ridges in the scapular region and two oblique ridges behind the eyes were visible in all examined specimens. In four specimens, they formed a chevron in the scapular region, in three specimens the ridges jointed laterally, forming an hourglass, and in two specimens all four ridges were disjointed.

Males and females differ in size and in proportions (Figure 2). Finger and toe tips are enlarged in females and fingertips are slightly enlarged in males. Males’ first finger is short and swollen but no distinct nuptial pad is visible. Adult males present femoral glands, which are lacking in females. Bicolor eggs are visible through the skin of gravid females.

Coloration of the body varies from golden to light brown, with few large light green blotches in some individuals. Most specimens present a dark chevron in the scapular region underlining two short back ridges that are either jointed or disjointed with two oblique ridges between the back of the eye and the shoulders. A more or less pronounced dark stripe is present between the nostril and the eye, and between the eye and the arm. A more or less distinctive dark bar is present between the eyes and some individuals present a lighter or green snout. The flanks and the sides of the head present numerous small white spots, extending to the upper lip in some individuals. The thighs and legs of some individuals are very faintly and irregularly banded. The back of the thighs is cream with small light grey spots. Vocal sac in adult males is white with the frontal third of the throat light grey with white freckles, while in females the throat and the ventral skin is light grey or yellowish molted with white. Some individuals have a thin light line on the backside of the thigh from the vent to the tarsal tubercle. A dark triangle is generally present in the back of the thigh, around the vent.

#### Etymology.

The specific name refers to Bibita Mountain, the type and only known locality for the species. It is an invariable noun used in apposition.

#### Habitat, distribution, and natural history.

**Figure 3.**
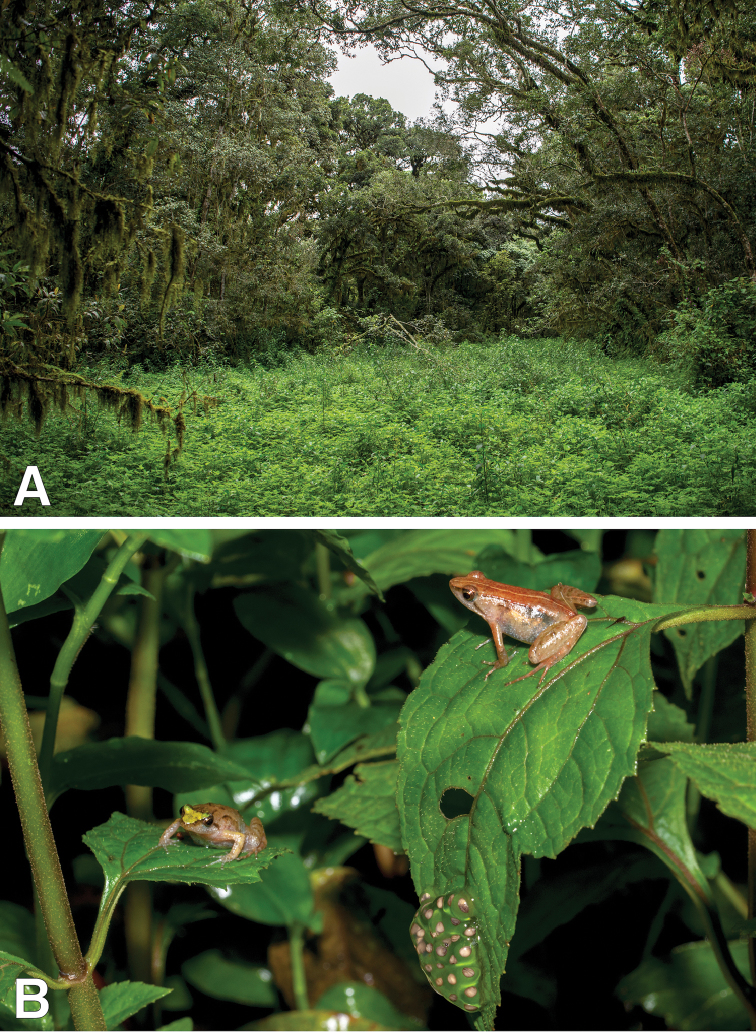
Habitat of *Phrynobatrachusbibita* sp. n. **A** Type locality of *P.bibita* sp. n. Overgrown pond in primary forest **B** Two females *P.bibita* sp. n. *in situ*, next to a clutch of eggs, in vegetation at ca. 30 cm above the water. Multiple females and egg clutches were found in similar circumstances.

All individuals were collected in a single large overgrown forest pond (Figure 3A), at night. The surrounding forest consisted of large trees with overhanging epiphytes and dense undergrowth. All females and the amplected pair were found on vegetation ca. 30 cm above water (Figure 3B). A single male was found in the water, presumably while calling. All collected females were gravid, and bicolor eggs were visible through the skin. Females seemed to aggregate in specific areas of the pond, were numerous egg clutches were found on leaves overhanging the water (Figure 3B). Laying eggs on vegetation overhanging the water is unusual in *Phrynobatrachus*, most species laying their eggs directly in the water (Zimkus et al. 2012). We thus confirmed that these eggs belonged to *Phrynobatrachusbibita* sp. n. by sequencing their mitochondrial rRNA 16s. Various forms of terrestrial egg deposition have been described in the genus *Phrynobatrachus* (Zimkus et al. 2012): most similarly to *P.bibita*, *P.sandersoni* (Parker, 1935) lays its eggs on vegetation up to 2 m above small puddles, small streams or water-saturated soil (Amiet 1981) and *P.krefftii* lays its eggs above the water, on rocks or vegetation (Harper and Vonesh 2010). *Phrynobatrachusguineensis* Guibé & Lamotte, 1961 lays its eggs on the bark of trees above water-filled tree holes (Rödel 1998) and *P.dendrobates* lays its eggs in tree holes or above streams (Zimkus et al. 2012). Finally, *P.phyllophilus* Rödel & Ernst, 2002, *P.tokba* (Chabanaud, 1921), and *P.villiersi* Guibé, 1969 lay their eggs on the leaf litter or the forest floor (Rödel and Ernst 2002a, 2002b; Zimkus et al. 2012). *Phrynobatrachusbibita* sp. n. thus adds to the diversity of reproductive modes in the genus.

Five egg clutches were photographed and contained approximately 30 eggs each (range 22 to 33). All observed clutches were found between 20 – 40 cm above water on vegetation, and up to two clutches were found on a single leaf. When laid, the eggs are bicolor, heavily pigmented, and encased in a thin gelatinous layer; as the egg develops, the pigmentation is more evenly distributed at its surface, with dark brown freckle, and the gelatinous layer becomes much thicker. It is possible that female *P.bibita* sp. n. are guarding their egg clutches in a similar manner as *P.sandersoni* females, which attend their eggs at night by standing over them or staying in the close proximity (Amiet 1981). Additional behavioral observations are necessary to determine whether females *P.bibita* sp. n. truly display such parental care. Other frog species were found in the same pond or nearby, including *Afrixalusenseticola* Largen, 1974, *Leptopelisvannutellii* (Boulenger, 1898), *Paracassinaobscura* (Boulenger, 1895), and Ptychadenacf.erlangeri (Ahl, 1924).

The forest in Bibita Mountain appears to be well preserved, as there are no settlements inside the forest, and a footpath is the only way to cross the forest. People from the surrounded villages harvest wild honey from the forest, but this is the only noticeable human activity there. Based on satellite imagery, no human disturbance is apparent at the higher elevations of the mountain; however, most areas below 1900 m have been transformed into agricultural land.

#### Call.

**Figure 4.**
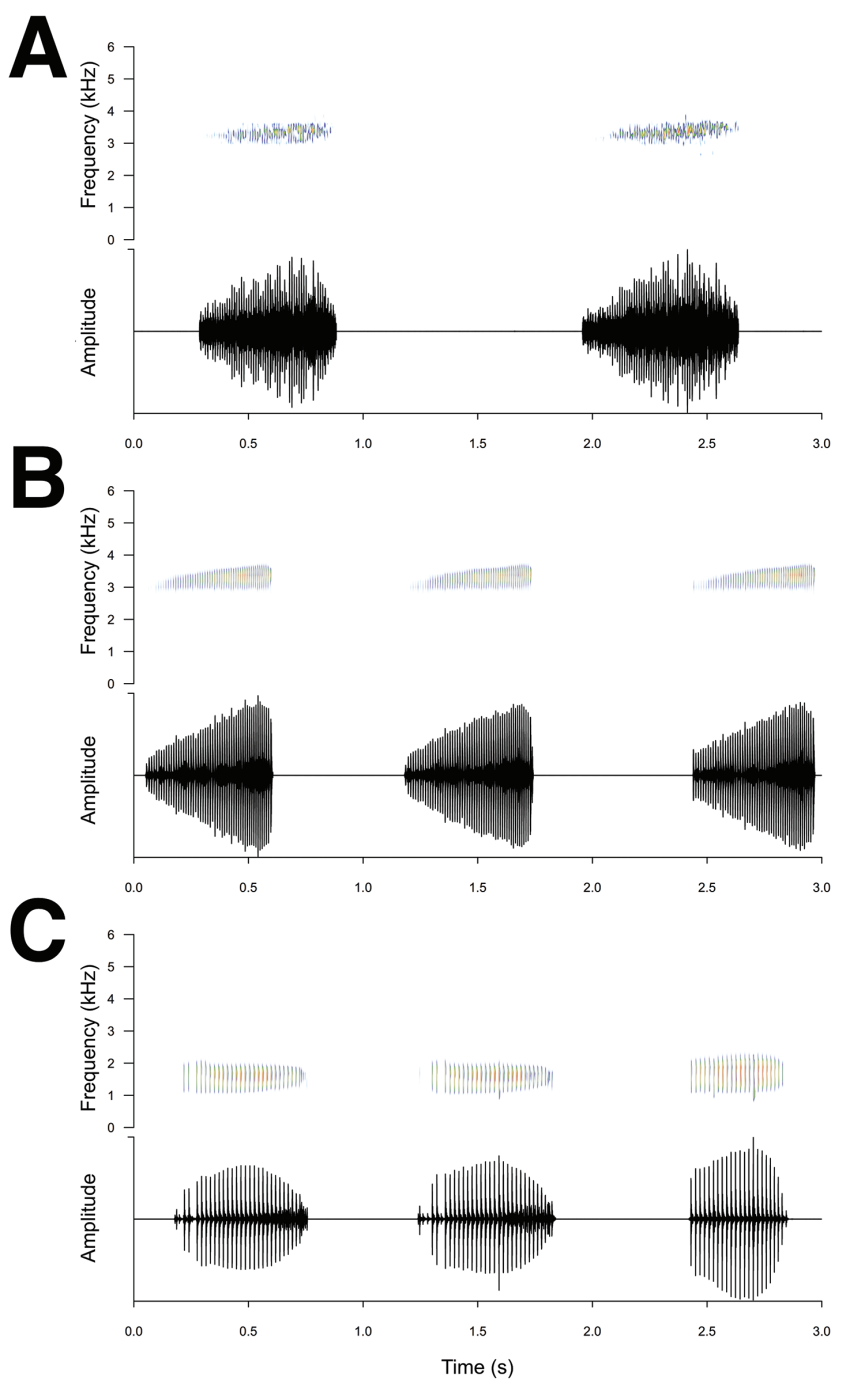
Advertisement call of *Phrynobatrachusbibita* sp. n. Spectrograms (upper panels) and sonograms (lower panels; relative amplitude) of Ethiopian *Phrynobatrachus* advertisement calls. **A***Phrynobatrachusbibita* sp. n. (specimen not collected) **B***P.minutus* (SB233) **C***P.natalensis* (specimen not collected).

The advertisement call is composed of a series of pulsed notes with a slight upward frequency modulation within each note (Figure 4A). It is similar to that of *Phrynobatrachusminutus* (Figure 4B) in spectral structure, note length, and dominant frequency. The vocalization of *P.bibita* sp. n. can be distinguished from that of *P.natalensis* (Figure 4C) by its higher dominant frequency, indicative of its smaller body size. Dominant frequency: 3318 ± 94 Hz. Note duration = 630 ± 80 ms. Interval inter-notes = 1085 ± 630 ms. The mediocre quality of our recording prevents us from analyzing the advertisement call of *P.bibita* sp. n. in further details, and a more complete description of their vocalization will be needed in the future, in particular to distinguish it from other species of dwarf *Phrynobatrachus*.

#### Estimates of evolutionary relationships.

**Figure 5.**
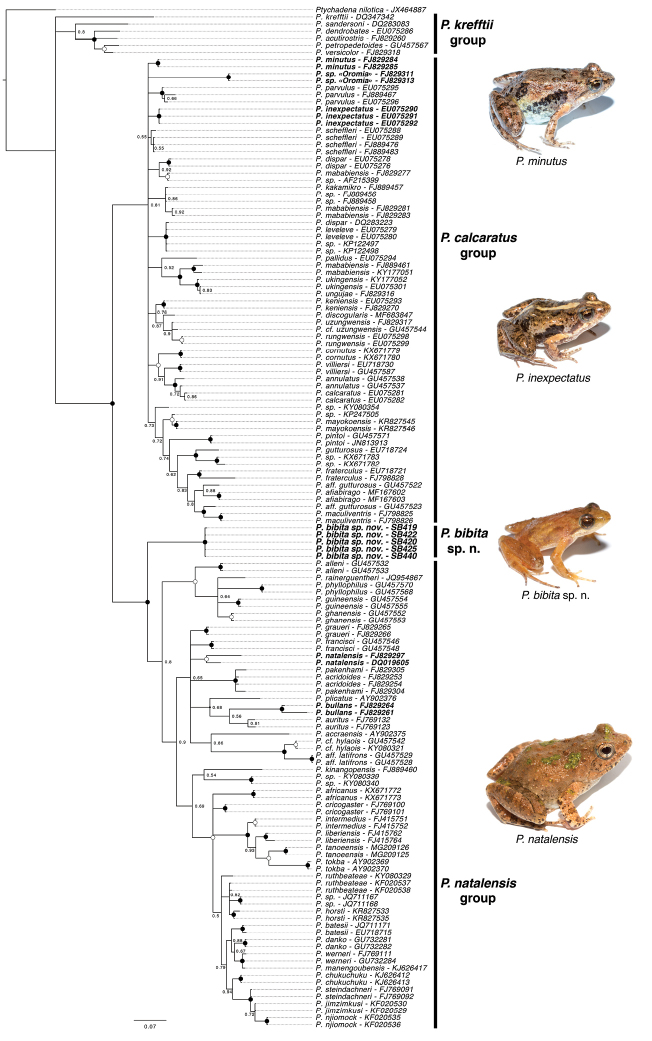
Phylogenetic placement of *Phrynobatrachusbibita* sp. n. Bayesian phylogenetic inference of the genus *Phrynobatrachus* based on the mitochondrial rRNA 16s. Nodes with a posterior support of 1 are marked with a black circle and nodes with high posterior support (>0.95) are marked with a white circle. Individuals of *Phrynobatrachus* species known to occur in Ethiopia are shown in boldface. Photos of Ethiopian representatives are displayed, from top to bottom: *Phrynobatrachusminutus* (SB175; Kibre Mengist), *P.inexpectatus* (SB143; Magnete, Harenna forest), *P.bibita* sp. n. (SB440; male holotype), *P.natalensis* (SB454; Mizan Teferi).

The goal of our phylogenetic analysis was to test the phylogenetic position of the new species, not to obtain a phylogeny of the genus, which has been previously done elsewhere (Zimkus et al. 2010). Our phylogenetic estimates recovered three main lineages in the genus *Phrynobatrachus* (Figure 5); however, several of the nodes have low support. We briefly describe the resulting topology below.

We recovered a basal group of *Phrynobatrachus*, which included the species *P.acutirostris* Nieden, 1912, *P.krefftii*, *P.dendrobates*, *P.petropedetoides* Ahl, 1924, and *P.sandersoni*. This clade is consistent with the “Clade A” of Zimkus et al. (2010), however this clade received low support (posterior probability (pp) < 0.5). All remaining species in the genus were grouped into two major clades, which received strong support (pp = 1). The first one of these two groups is similar to the “Clade B” of Zimkus et al. (2010), which includes the Ethiopian species *P.minutus*, *P.inexpectatus*, and *P.* sp. n. “Oromia” of Zimkus and Schick (2010), while the second group is consistent with the “Clade C” of Zimkus et al. (2010), and includes the Ethiopian species *P.natalensis*. *Phrynobatrachusbibita* sp. n. was not closely related to any of the other Ethiopian species, and instead basal to the last group (“Clade C” of Zimkus et al. (2010)), however with high support (pp = 1). Our analysis grouped the egg clutch collected (SB422) with the adults *P.bibita* sp. n. (holotype SB440 and paratopotypes SB419, SB420, SB425), ensuring that it belonged to the new species. We did not include samples of three junior synonyms of *P.natalensis* from Ethiopia (see *Comparisons*), however, samples referable to these junior synonyms have previously been included on a phylogeny of members of the *P.natalensis* group (Lara 2016), and they are well nested within *P.natalensis*, we can thus conclude that they do not represent *P.bibita*.

## Discussion

Most of the natural vegetation of the Ethiopian highlands has been transformed for agriculture or into grazing fields for cattle (Williams et al. 2004). However, in the southwestern end of the country, a few areas still conserve their original vegetation, and several of these areas remain largely unexplored. Bibita Mountain is one of such areas, and to our knowledge, no vertebrate collections has ever been obtained there. The only published account on the flora or fauna of Bibita Mountain comes from a study on plant composition and structure (Hundera and Deboch 2008). The discovery of a distinctly new species of *Phrynobatrachus* after only two days of survey calls out for further exploration of the biological diversity in this area. Our previous research showed that the amphibian fauna of Ethiopia is underestimated by the current taxonomy, and we believe that further research on Bibita Mountain, as well as in other nearby areas might greatly increase the number of species of amphibians in Ethiopia.

The phylogenetic relationships of many members of the genus *Phrynobatrachus* remains unresolved. Zimkus et al. (2010) recovered three main clades of *Phrynobatrachus* in their analysis, which they designated as groups ‘A’, ‘B’ and ‘C’. Our results show that *P.bibita* sp. n. is not related to other currently described species of Ethiopian *Phrynobatrachus*, and instead, it appears to be sister to all the members of the *P.natalensis* group (Figure 5). Interestingly, the morphology of *P.bibita* sp. n., including long limbs, elongated fingers and toes, enlarged toe tips and pointed snout, is most similar to the more distantly related species *P.sandersoni*, *P.dendrobates*, and *P.krefftii*. In addition, *P.bibita* sp. n. shares with these species a rare behavior for the genus consisting of laying eggs on vegetation above the water (Amiet 1981, Harper and Vonesh 2010, Zimkus et al. 2012). Finally, *P.bibita* sp. n. might display parental care, as observed in *P.sandersoni* and *P.dendrobates* (Amiet 1981, Zimkus et al. 2012) and unknown from any other species of the genus. Whether *P.bibita* sp. n. retained ancestral morphological and behavioral characters shared with the *P.krefftii* species group, or whether it is a case of convergence remains to be investigated. Additional work is necessary to better understand the evolutionary history of this diverse genus.

## Supplementary Material

XML Treatment for Phrynobatrachus bibita

## Supplementary materials

Supplementary material 1 Table S1. GenBank accession numbers Data type: molecular data This is an open access article distributed under the terms of the Creative Commons Attribution License (CC BY 4.0), which permits unrestricted use, distribution, and reproduction in any medium, provided the original author and source are credited.Sandra Goutte, Jacobo Reyes-Velasco, Stephane Boissinot

Supplementary material 2 Photographs of some morphological details of *Phrynobatrachusbibita* sp. n. Data type: multimedia Explanation note: A – Right foot of female paratopotype SB424, showing the tarsal tubercle (a), inner metatarsal tubercle (b) and outer metatarsal tubercle (c). B – Left hind limb of male holotype SB440, showing the femoral gland. This is an open access article distributed under the terms of the Creative Commons Attribution License (CC BY 4.0), which permits unrestricted use, distribution, and reproduction in any medium, provided the original author and source are credited.Sandra Goutte, Jacobo Reyes-Velasco, Stephane Boissinot
