# Supplementary material for: A new species of puddle frog from an unexplored mountain in southwestern Ethiopia (Anura, Phrynobatrachidae, Phrynobatrachus)
Source: Zookeys. 2019 Feb 12;(824):53–70. doi: 10.3897/zookeys.824.31570 (PMC6381079; doi:10.3897/zookeys.824.31570)
Supplement: Supplementary material 1 [file zookeys-824-053-s001.pdf]

Table S1. GenBank accession numbers.

Genus	species	Genbank number	Field number	Notes
Phrynobatrachus	*bibita* sp. n.	MK139950	SB419	Paratopotype
Phrynobatrachus	*bibita* sp. n.	MK139951	SB422	Paratopotype
Phrynobatrachus	*bibita* sp. n.	MK139952	SB420	Paratopotype
Phrynobatrachus	*bibita* sp. n.	MK139953	SB425	Paratopotype
Phrynobatrachus	*bibita* sp. n.	MK139954	SB440	Holotype
Phrynobatrachus	*accraensis*	AY902375	-	-
Phrynobatrachus	*acridoides*	FJ829253	-	-
Phrynobatrachus	*acridoides*	FJ829254	-	-
Phrynobatrachus	*acutirostris*	FJ829260	-	-
Phrynobatrachus	aff. *gutturosus*	GU457522	-	-
Phrynobatrachus	aff. *gutturosus*	GU457523	-	-
Phrynobatrachus	aff. *latifrons*	GU457528	-	-
Phrynobatrachus	aff. *latifrons*	GU457529	-	-
Phrynobatrachus	*afiabirago*	MF167602	-	-
Phrynobatrachus	*afiabirago*	MF167603	-	-
Phrynobatrachus	*africanus*	KX671772	-	-
Phrynobatrachus	*africanus*	KX671773	-	-
Phrynobatrachus	*alleni*	GU457532	-	-
Phrynobatrachus	*alleni*	GU457533	-	-
Phrynobatrachus	*annulatus*	GU457537	-	-
Phrynobatrachus	*annulatus*	GU457538	-	-
Phrynobatrachus	*auritus*	FJ769123	-	-
Phrynobatrachus	*auritus*	FJ769132	-	-
Phrynobatrachus	*batesii*	EU718715	-	-
Phrynobatrachus	*batesii*	JQ711171	-	-
Phrynobatrachus	*bullans*	FJ829261	-	-
Phrynobatrachus	*bullans*	FJ829264	-	-
Phrynobatrachus	*calcaratus*	EU075281	-	-
Phrynobatrachus	*calcaratus*	EU075282	-	-
Phrynobatrachus	cf. *hylaois*	GU457542	-	-
Phrynobatrachus	cf. *hylaois*	KY080321	-	-
Phrynobatrachus	cf. *uzungwensis*	GU457544	-	-
Phrynobatrachus	*chukuchuku*	KJ626412	-	-
Phrynobatrachus	*chukuchuku*	KJ626413	-	-
Phrynobatrachus	*cornutus*	KX671779	-	-
Phrynobatrachus	*cornutus*	KX671780	-	-
Phrynobatrachus	*cricogaster*	FJ769100	-	-
Phrynobatrachus	*cricogaster*	FJ769101	-	-
Phrynobatrachus	*danko*	GU732281	-	-
Phrynobatrachus	*danko*	GU732282	-	-
Phrynobatrachus	*dendrobates*	EU075286	-	-
Phrynobatrachus	*discogularis*	MF683847	-	-
Phrynobatrachus	*dispar*	DQ283223	-	-

Genus	species	Genbank number	Field number	Notes
Phrynobatrachus	*dispar*	EU075276	-	-
Phrynobatrachus	*dispar*	EU075278	-	-
Phrynobatrachus	*francisci*	GU457546	-	-
Phrynobatrachus	*francisci*	GU457548	-	-
Phrynobatrachus	*fraterculus*	EU718721	-	-
Phrynobatrachus	*fraterculus*	FJ798828	-	-
Phrynobatrachus	*ghanensis*	GU457552	-	-
Phrynobatrachus	*ghanensis*	GU457553	-	-
Phrynobatrachus	*graueri*	FJ829265	-	-
Phrynobatrachus	*graueri*	FJ829266	-	-
Phrynobatrachus	*guineensis*	GU457554	-	-
Phrynobatrachus	*guineensis*	GU457555	-	-
Phrynobatrachus	*gutturosus*	EU718724	-	-
Phrynobatrachus	*horsti*	KR827533	-	-
Phrynobatrachus	*horsti*	KR827535	-	-
Phrynobatrachus	*inexpectatus*	EU075290	-	-
Phrynobatrachus	*inexpectatus*	EU075291	-	-
Phrynobatrachus	*inexpectatus*	EU075292	-	-
Phrynobatrachus	*intermedius*	FJ415751	-	-
Phrynobatrachus	*intermedius*	FJ415752	-	-
Phrynobatrachus	*jimzimkusi*	KF020529	-	-
Phrynobatrachus	*jimzimkusi*	KF020530	-	-
Phrynobatrachus	*kakamikro*	FJ889457	-	-
Phrynobatrachus	*keniensis*	EU075293	-	-
Phrynobatrachus	*keniensis*	FJ829270	-	-
Phrynobatrachus	*kinangopensis*	FJ889460	-	-
Phrynobatrachus	*krefftii*	DQ347342	-	-
Phrynobatrachus	*leveleve*	EU075279	-	-
Phrynobatrachus	*leveleve*	EU075280	-	-
Phrynobatrachus	*liberiensis*	FJ415762	-	-
Phrynobatrachus	*liberiensis*	FJ415764	-	-
Phrynobatrachus	*mababiensis*	FJ829277	-	-
Phrynobatrachus	*mababiensis*	FJ829281	-	-
Phrynobatrachus	*mababiensis*	FJ829283	-	-
Phrynobatrachus	*mababiensis*	FJ889461	-	-
Phrynobatrachus	*mababiensis*	KY177051	-	-
Phrynobatrachus	*maculiventris*	FJ798825	-	-
Phrynobatrachus	*maculiventris*	FJ798826	-	-
Phrynobatrachus	*manengoubensis*	KJ626417	-	-
Phrynobatrachus	*mayokoensis*	KR827545	-	-
Phrynobatrachus	*mayokoensis*	KR827546	-	-
Phrynobatrachus	*minutus*	FJ829284	-	-
Phrynobatrachus	*minutus*	FJ829285	-	-
Phrynobatrachus	*natalensis*	DQ019605	-	-

Genus	species	Genbank number	Field number	Notes
Phrynobatrachus	*natalensis*	FJ829297	-	-
Phrynobatrachus	*njiomock*	KF020535	-	-
Phrynobatrachus	*njiomock*	KF020536	-	-
Phrynobatrachus	*pakenhami*	FJ829304	-	-
Phrynobatrachus	*pakenhami*	FJ829305	-	-
Phrynobatrachus	*parvulus*	EU075295	-	-
Phrynobatrachus	*parvulus*	EU075296	-	-
Phrynobatrachus	*petropedetoides*	GU457567	-	-
Phrynobatrachus	*phyllophilus*	GU457568	-	-
Phrynobatrachus	*phyllophilus*	GU457570	-	-
Phrynobatrachus	*pallidus*	EU075294	-	-
Phrynobatrachus	*pintoi*	GU457571	-	-
Phrynobatrachus	*pintoi*	JN813913	-	-
Phrynobatrachus	*plicatus*	AY902376	-	-
Phrynobatrachus	*rainerguentheri*	JQ954867	-	-
Phrynobatrachus	*rungwensis*	EU075298	-	-
Phrynobatrachus	*rungwensis*	EU075299	-	-
Phrynobatrachus	*ruthbeateae*	KF020537	-	-
Phrynobatrachus	*ruthbeateae*	KF020538	-	-
Phrynobatrachus	*ruthbeateae*	KY080329	-	-
Phrynobatrachus	*sandersoni*	DQ283083	-	-
Phrynobatrachus	*scheffleri*	FJ889476	-	-
Phrynobatrachus	*scheffleri*	FJ889483	-	-
Phrynobatrachus	sp.	KP247505	-	-
Phrynobatrachus	sp.	KX671782	-	-
Phrynobatrachus	sp.	KX671783	-	-
Phrynobatrachus	sp.	KY080339	-	-
Phrynobatrachus	sp.	KY080340	-	-
Phrynobatrachus	sp.	AF215399	-	-
Phrynobatrachus	sp. nov. “Oromia”	FJ829311	-	-
Phrynobatrachus	sp. nov. “Oromia”	FJ829313	-	-
Phrynobatrachus	sp.	FJ889456	-	-
Phrynobatrachus	sp.	FJ889458	-	-
Phrynobatrachus	sp.	JQ711167	-	-
Phrynobatrachus	sp.	JQ711168	-	-
Phrynobatrachus	sp.	KP122497	-	-
Phrynobatrachus	sp.	KP122498	-	-
Phrynobatrachus	sp.	KY080354	-	-
Phrynobatrachus	*steindachneri*	FJ769091	-	-
Phrynobatrachus	*steindachneri*	FJ769092	-	-
Phrynobatrachus	*tanoeensis*	MG209125	-	-
Phrynobatrachus	*tanoeensis*	MG209126	-	-
Phrynobatrachus	*tokba*	AY902369	-	-
Phrynobatrachus	*tokba*	AY902370	-	-

Genus	species	Genbank number	Field number	Notes
Phrynobatrachus	*ukingensis*	EU075301	-	-
Phrynobatrachus	*ukingensis*	KY177052	-	-
Phrynobatrachus	*ungujae*	FJ829316	-	-
Phrynobatrachus	*uzungwensis*	FJ829317	-	-
Phrynobatrachus	*versicolor*	FJ829318	-	-
Phrynobatrachus	*villiersi*	EU718730	-	-
Phrynobatrachus	*villiersi*	GU457587	-	-
Phrynobatrachus	*werneri*	FJ769111	-	-
Phrynobatrachus	*werneri*	GU732284	-	-
Ptychadena	*nilotica*	JXR464887	-	Outgroup
