# Supplementary figures and images for: A new species of puddle frog from an unexplored mountain in southwestern Ethiopia (Anura, Phrynobatrachidae, Phrynobatrachus)
Source: Zookeys. 2019 Feb 12;(824):53–70. doi: 10.3897/zookeys.824.31570 (PMC6381079; doi:10.3897/zookeys.824.31570)

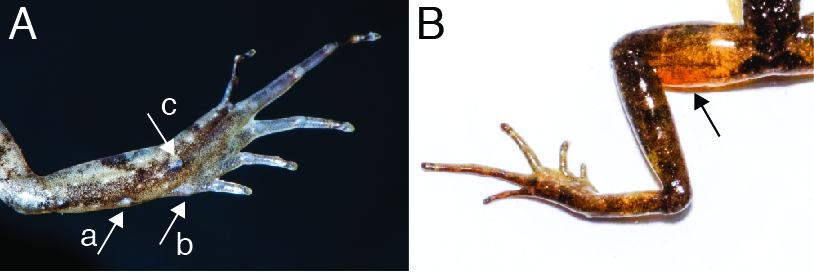

Supplement: Supplementary material 2 [file zookeys-824-053-s002.jpg]
